# Treatment of acute rhinitis with a nasal spray containing tramazoline and essential oils: a multicenter, uncontrolled, observational trial

**DOI:** 10.1186/s13601-015-0084-5

**Published:** 2015-11-04

**Authors:** Gábor Katona, Mónika Sultész, Zsolt Farkas, Andrea Gyimesi, Andor Hirschberg, János Huszka, Ferenc Radvánszky, Ákos Simon, Gabriella Fülöp, Júlia Láng, Mária Ablonczy, Günther Nirnberger, Claudia Holm

**Affiliations:** ENT Department, Heim Pál Children’s Hospital, Üllői U.86., 1089 Budapest, Hungary; ENT Department, St. John’s Hospital, Budapest, Hungary; ENT Department, Péterfy Str. Hospital, Budapest, Hungary; ENT Department, Second Dept of Pediatrics, Semmelweis University, Budapest, Hungary; Bioconsult GmbH, Fritz Konir-Gasse 19, 2384 Breitenfurt, Austria; Boehringer Ingelheim RCV GmbH & Co. KG, Dr. Boehringer-Gasse 5-11, 1121 Vienna, Austria

## Abstract

**Background:**

In this observational trial, data were collected on the effectiveness and tolerability/safety of a nasal spray containing tramazoline and essential oils (trade name Rhinospray^®^ Plus) used for symptomatic treatment of acute rhinitis due to common cold.

**Methods:**

The trial was performed in 300 children, adolescents and adults, who were to be treated with Rhinospray^®^ Plus for up to 4 times per day for up to 10 days. Primary endpoints were the change from baseline to final visit in the mean of three single symptom scores (blocked nose, sneezing, and runny nose) and the mean improvement in two quality-of-life parameters (ability to perform normal daytime activities and quality of sleep).

**Results:**

A total of 108 children, 30 adolescents and 162 adults were treated with Rhinospray^®^ Plus. No patient discontinued prematurely. There was a mean reduction of 2.0 ± 0.6 (standard deviation) in nasal symptom scores from baseline to final visit; 297 of 300 of patients (99.0 %) reported an improvement. The mean value for improvement in quality-of-life parameters was 1.3 ± 0.5. Improvement in daytime activities was reported by all 300 patients (100.0 %) and in quality of sleep by 292 patients (97.4 %). Effectiveness and tolerability were rated as ‘very good’ or ‘good’ by 95.4 % and 97.4 % of patients, respectively; the investigators rated effectiveness and tolerability as ‘very good’ or ‘good’ for 97.4 % and 100.0 % of patients, respectively. No adverse events were reported.

**Conclusions:**

Community-based patients reported a relief in acute rhinitis symptoms and improvement in quality of life as a result of treatment with Rhinospray^®^ Plus. Treatment was well-tolerated.

## Findings

### Background

A troublesome feature of common cold is acute rhinitis (inflammation of the nasal mucous membranes resulting in congestion, rhinorrhoea, and sneezing). Nasal congestion negatively impacts daily activities and is a common complaint in children and adults who visit otorhinolaryngologists [1]. Congestion can be treated with topical nasal sprays containing imidazoline derivatives [1], which stimulate alpha-adrenergic receptors and induce vasoconstriction in the nose and paranasal sinuses, thereby reducing swelling and mucus production.

Rhinospray^®^ Plus is a topical nasal spray containing tramazoline hydrochloride monohydrate and essential oils (eucalyptol, levomenthol and camphor); it is effective and well-tolerated in treatment of nasal congestion caused by acute rhinitis due to common cold or hay fever [1–5]. Rhinospray^®^ Plus is suitable for adults and children 6 years of age and older. The recommended dose is a single puff of spray (0.07 mL spray containing 0.09 mg tramazoline) into each nostril, up to 4 times daily for up to 7 days [6]. Based on available data and clinical experience, however, treatment durations of 10–15 days are acceptable.

The goal of this observational study was for patients and investigators to assess effectiveness and tolerability/safety of Rhinospray^®^ Plus treatment of acute nasal congestion caused by common cold. The study was conducted in a community (real-life) setting, with emphasis on quality-of-life measures.

## Methods

Patients who presented with cold symptoms to one of four outpatient centres, received a clinical diagnosis of acute rhinitis, and had not previously used Rhinospray^®^ Plus were eligible for enrolment. Patients provided informed consent (for minors, this was provided by a parent/guardian). Exclusion criteria included allergic rhinitis, contraindications to Rhinospray^®^ Plus, participation in another trial, pregnancy/breastfeeding, or inability to cooperate.

Patients underwent two ambulatory clinic visits, at baseline and on the day after treatment was terminated. At the baseline visit, patients rated three nasal symptoms (blocked nose, sneezing and runny nose) on a 0–3 scale (0 = absent, 1 = mild, 2 = moderate, 3 = severe) [7]. Treatment with Rhinospray^®^ Plus was to start on the same day as the baseline visit. Patients were instructed to use one puff of Rhinospray^®^ Plus per nostril up to 4 times daily for up to 10 days. They also received a diary in which to record medication use, onset and duration of action of Rhinospray^®^ Plus, and daily nasal symptom scores.

At the final visit, patients rated nasal symptoms and improvement in two quality-of-life parameters (ability to perform normal daytime activities and quality of sleep) on a 1–4 scale (1 = strong improvement, 2 = moderate improvement, 3 = weak improvement, 4 = no improvement), as well as overall effectiveness and tolerability on a 1–4 scale (1 = very good, 2 = good, 3 = fair, 4 = poor). The patients also answered a questionnaire assessing comfort of application and effect of the essential oils. Investigators recorded their assessments of effectiveness and tolerability and patients’ spontaneous reporting of adverse events.

Primary endpoints were the change from baseline to final visit in the mean of nasal symptom scores and the mean improvement in quality-of-life parameters. Secondary endpoints were the change from baseline to final visit in single nasal symptom scores, the improvement in each quality-of-life parameter, and the patients’ and investigators’ assessment of treatment effectiveness. Tolerability/safety was evaluated on the basis of adverse events and patients’ and investigators’ assessment of tolerability. Additional parameters included onset of action and duration of the treatment effect, and evaluation of comfort of application and the essential oil component of the spray. Data were analysed with descriptive statistics. Safety analyses included all treated patients and effectiveness analyses included all patients for whom a final visit was recorded. The study was approved by the Medical Research Council, Scientific and Research Committee, Reference number: 40683-2/2013/EKU (482/2013), by the Hungarian Regulatory Authority (Reference number: OGYI/30633-6/2013) and was registered in the clinicaltrials.gov database under the identifier NCT01971086.

## Results

Three hundred patients were enrolled and treated; for all patients, a final visit was recorded. No patient discontinued treatment. Of the 300 patients, 162 (54.0 %) were adults (age 18 and over), 30 (10.0 %) were adolescents (age 13–17) and 108 (36.0 %) were children (age 6–12). Additional patient characteristics are provided in Table 1.

**Table 1:** Demographics and baseline characteristics, treated set

	Children	Adolescents	Adults	Total
Patients, N (%)	108 (36.0)	30 (10.0)	162 (54.0)	300 (100)
Mean age in years (SD)	8.4 (2.0)	14.6 (1.3)	42.1 (15.9)	27.2 (20.0)
Sex, N (%)				
Female	37 (34.3)	15 (50.0)	94 (58.0)	146 (48.7)
Male	71 (65.7)	15 (50.0)	68 (42.0)	154 (51.3)
Height (SD)	134 (15.1)	167 (16.9)	173 (9.8)	158 (22.1)
Weight (SD)	31.8 (10.2)	59.6 (16.1)	75.3 (18.2)	58 (25.5)
Mean days since symptom onset (SD)	1.3 (1.2)	1.1 (1.9)	1.4 (1.9)	1.3 (1.7)
Baseline mean symptom score (SD)	2.1 (0.6)	2.1 (0.6)	2.1 (0.6)	2.1 (0.6)
Pretreated with other medications, N (%)	8 (7.4)	6 (20.0)	30 (18.5)	44 (14.7)
Concomitant diagnosis, N (%)^a^	48 (44.4)	8 (26.7)	45 (27.8)	101 (33.7)
Ear and labyrinth disorders	22 (20.4)	4 (13.3)	5 (3.1)	31 (10.3)
Respiratory, thoracic and mediastinal disorders	21 (19.4)	2 (6.7)	3 (1.9)	26 (8.7)
Vascular disorders	0 (0.0)	0 (0.0)	18 (11.1)	18 (6.0)
Gastrointestinal disorders	4 (3.7)	0 (0.0)	13 (8.0)	17 (5.7)
Concomitant medications, N (%)^b^	49 (45.4)	7 (23.3)	45 (27.8)	101 (33.7)
Antibiotics, N (%)	31 (28.7)	3 (10.0)	4 (2.5)	38 (12.7)
Smoker, N (%)	0 (0.0)	0 (0.0)	23 (14.2)	23 (7.7)

^a^Concomitant diagnosis is listed only for those categories including >5 % of patients

^b^Concomitant medications are listed only for those categories including >5 % of patients

### Primary endpoints

**Fig. 1:**
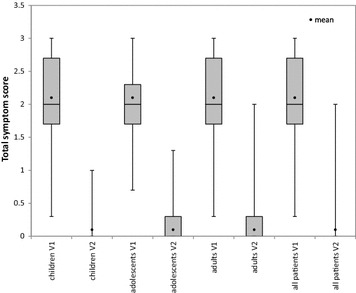
Quartile scores, median, minimum and maximum scores, and mean values for the total of the three nasal symptoms at Visit 1 (V1) and Visit 2 (V2), full analysis set. For children and for all patients at Visit 2, quartile 1, median, and quartile 3 values were zero

Including all patients, the mean symptom score was 2.1 ± 0.6 at baseline and 0.1 ± 0.3 at the final visit; the mean decrease was 2.0 ± 0.7 (Table 2). Of the 300 patients, 297 (99.0 %) reported a decrease in mean symptom score. The median symptom score was 0 at the final visit (Fig. 1). The mean improvement in the quality of life parameters was 1.3 ± 0.5 (Table 2), and all patients (100 %) reported an improvement on at least one quality-of-life measure.

**Table 2:** Primary endpoints, full analysis set

	Children	Adolescents	Adults	Total
Patients, N (%)	108 (36.0)	30 (10.0)	162 (54.0)	300 (100)
Symptom score of three nasal indices				
Mean score at final visit (SD)	0.1 (0.3)	0.1 (0.3)	0.1 (0.2)	0.1 (0.3)
Mean change from first visit (SD)	−1.9 (0.7)	−1.9 (0.7)	−2.0 (0.6)	−2.0 (0.7)
Median score at final visit (min, max)	0 (0, 1.0)	0 (0, 1.3)	0 (0, 2)	0 (0, 2)
Median change from first visit (min, max)	−2 (−3, 0)	−2 (−3, 0.7)	−2 (−3, 0.7)	−2 (−3, 0.7)
Improvement in quality of life				
Mean improvement (SD)	1.2 (0.5)	1.2 (0.5)	1.3 (0.5)	1.3 (0.5)
Median improvement (min, max)	1 (1, 3)	1 (1, 3)	1 (1, 3)	1 (1, 3)

### Secondary endpoints

**Fig. 2:**
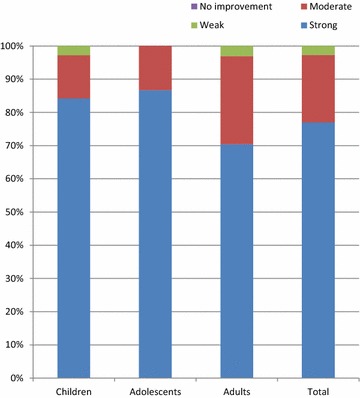
Improvement in daytime activities

**Fig. 3:**
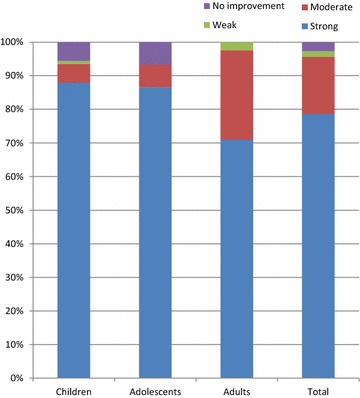
Improvement in quality of sleep

**Fig. 4:**
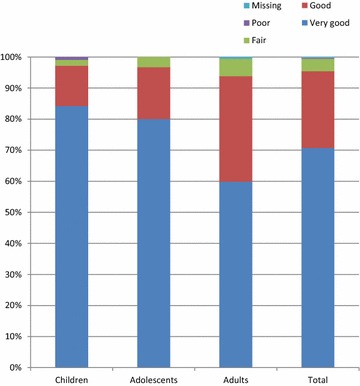
Patients’ assessment of effectiveness

At the final visit the mean blocked nose, sneezing and runny nose scores were 0.2 ± 0.4, 0.0 ± 0.2 and 0.1 ± 0.4, respectively; mean decreases were 2.4 ± 0.8, 1.4 ± 0.9 and 2.2 ± 0.8, respectively (Table 3). The median and third quartile scores were 0 for all nasal symptoms at the final visit. For improvement in daily activities, the mean score at final visit was 1.3 ± 0.5 and for improvement in sleep was 1.3 ± 0.6. Most patients reported a strong improvement in daytime activities (77.0 %) and in sleep quality (78.7 %; Figs. 2, 3). Overall, 97.3 % of patients rated the effectiveness of Rhinospray^®^ Plus as ‘very good’ or ‘good’ (Fig. 4), and 95.4 % of investigators rated the effectiveness as ‘very good’ or ‘good’ (Table 3).

**Table 3:** Secondary endpoints, full analysis set

	Children	Adolescents	Adults	Total
Patients, N (%)	108 (36.0)	30 (10.0)	162 (54.0)	300 (100)
Blocked nose				
Mean score at final visit (SD)	0.2 (0.5)	0.2 (0.5)	0.2 (0.4)	0.2 (0.4)
Mean change from first visit (SD)	−2.2 (0.9)	−2.2 (0.9)	−2.5 (0.7)	−2.4 (0.8)
Median score at final visit (min, max)	0 (0, 2)	0 (0, 2)	0 (0, 2)	0 (0, 2)
Median change from first visit (min, max)	−2 (−3, 1)	−2 (−3, 0)	−2 (−3, 0)	−2 (−3, 1)
Sneezing				
Mean score at final visit (SD)	0.1 (0.2)	0.1 (0.3)	0.0 (0.2)	0.0 (0.2)
Mean change from first visit (SD)	−1.4 (1.0)	−1.5 (0.9)	−2.0 (0.6)	−1.4 (0.9)
Median score at final visit (min, max)	0 (0, 1)	0 (0,1)	0 (0, 2)	0 (0, 2)
Median change from first visit (min, max)	−1 (−3, 0)	−1 (−3, 0)	−1 (−3, 2)	−1 (−3, 2)
Runny nose				
Mean score at final visit (SD)	0.1 (0.3)	0.2 (0.5)	0.1 (0.4)	0.1 (0.4)
Mean change from first visit (SD)	−2.2 (0.8)	−2.0 (0.8)	−2.1 (0.9)	−2.2 (0.8)
Median score at final visit (min, max)	0 (0, 2)	0 (0, 2)	0 (0, 2)	0 (0, 2)
Median change from first visit (min, max)	−2 (−3, 0)	−2 (−3, 0)	−2 (−3, 1)	−2 (−3, 1)
Improvement in daily activities				
Mean score at final visit (SD)	1.2 (0.5)	1.1 (0.3)	1.3 (0.5)	1.3 (0.5)
Median score at final visit (min, max)	1 (1, 3)	1 (1, 2)	1 (1, 3)	1 (1, 3)
Improvement in sleep				
Mean score at final visit (SD)	1.2 (0.7)	1.3 (0.8)	1.3 (0.5)	1.3 (0.6)
Median score at final visit (min, max)	1 (1, 4)	1 (1, 4)	1 (1, 3)	1 (1, 4)
Patients’ effectiveness assessment, N (%)				
Very good	95 (88.0)	24 (80.0)	103 (63.6)	222 (74.0)
Good	11 (10.2)	5 (16.7)	54 (33.3)	70 (23.3)
Fair	2 (1.9)	1 (3.3)	5 (3.1)	8 (2.7)
Poor	0 (0.0)	0 (0.0)	0 (0.0)	0 (0.0)
Investigators’ effectiveness assessment, N (%)				
Very good	91 (84.3)	24 (80.0)	97 (59.9)	212 (70.7)
Good	14 (13.0)	5 (16.7)	55 (34.0)	74 (24.7)
Fair	2 (1.9)	1 (3.3)	9 (5.6)	12 (4.0)
Poor	1 (0.9)	0 (0.0)	0 (0.0)	1 (0.3)
Missing	0 (0.0)	0 (0.0)	1 (0.6)	1 (0.3)

### Additional parameters

**Fig. 5:**
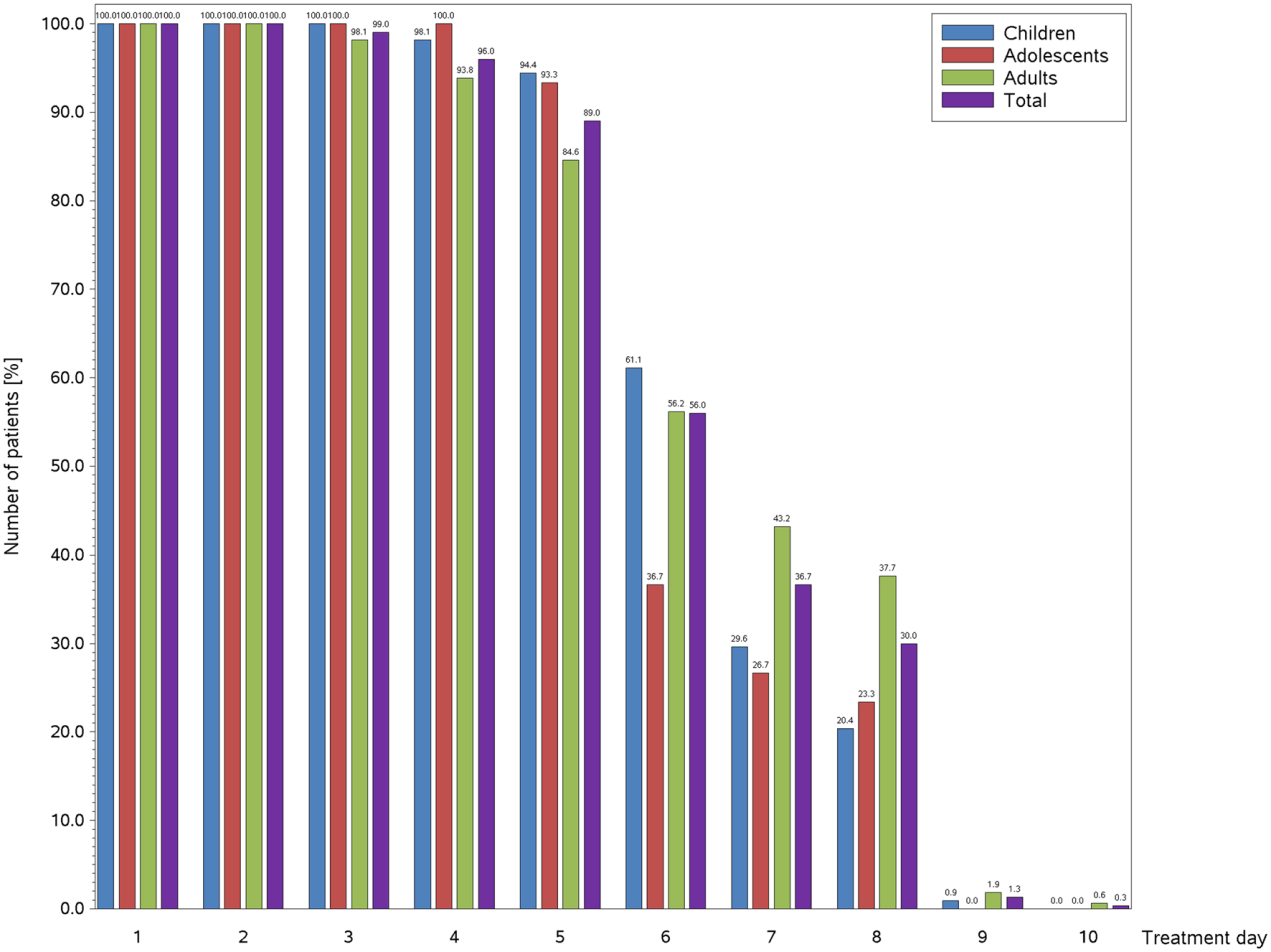
Summary of number of patients on treatment over time

The majority of patients (92.4 %) reported that treatment started to work within 5 min or fewer. More than half of patients (52.0 %) reported a duration of effect between 4 and 8 h (Table 4). For application comfort, 76.7 % of patients indicated it was comfortable as could be and 57.7 % stated that the spray did not flow into the larynx. For the essential oils, 56.7 % of patients indicated that they gave the spray a pleasant feeling, 52.0 % that they promoted free breathing and 48.7 % that they provided a clear, cool feeling. Patients’ diaries indicated a gradual improvement of symptoms over time, with only 36.7 % of patients still using Rhinospray^®^ Plus on day 7 (Fig. 5). On Day 7, 51.3 % of patients continued to reported nasal symptoms (51.2 % of adults, 53.7 % of children and 43.3 % of adolescents).

**Table 4:** Onset of treatment effect and duration of treatment effect, full analysis set

	Children	Adolescents	Adults	Total
Patients, N (%)	108 (36.0)	30 (10.0)	162 (54.0)	300 (100)
Time until treatment took effect, N (%)				
After less than 1 min	21 (19.4)	6 (20.0)	27 (16.4)	54 (18.0)
Between 1 and 3 min	59 (54.6)	15 (50.0)	63 (38.9)	137 (45.7)
Between 3 and 5 min	24 (22.2)	7 (23.3)	55 (34.0)	86 (28.7)
After more than 5 min	3 (2.8)	2 (6.7)	17 (10.5)	22 (7.3)
Information missing	1 (0.9)	0 (0.0)	0 (0.0)	1 (0.3)
Duration of treatment effect, N (%)				
Fewer than 4 h	30 (27.8)	7 (23.3)	57 (35.2)	94 (31.3)
Between 4 and 8 h	56 (51.9)	14 (46.7)	86 (53.1)	156 (52.0)
Between 8 and 10 h	21 (19.4)	8 (26.7)	17 (10.5)	46 (15.3)
More than 10 h	1 (0.9)	0 (0.0)	2 (1.2)	3 (1.0)
Information missing	0 (0.0)	1 (3.3)	0 (0.0)	1 (0.3)

### Safety endpoints

**Fig. 6:**
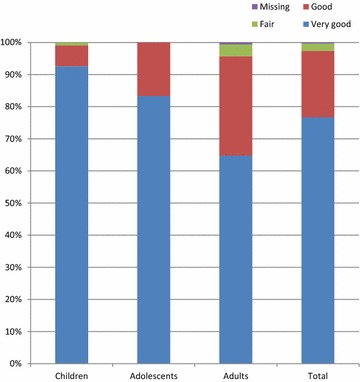
Patients’ assessment of tolerability

Mean duration of treatment was 7.1 days; there were no relevant differences based on patient age (Table 5). Overall, 97.7 % of patients (Fig. 6) and 100.0 % of investigators rated the treatment tolerability as ‘very good’ or ‘good’ (Table 5). There were no adverse events reported.

**Table 5:** Extent of exposure and tolerability assessments, treated set

	Children	Adolescents	Adults	Total
Patients, N (%)	108 (36.0)	30 (10.0)	162 (54.0)	300 (100)
Exposure to trial medication				
Mean days of exposure to trial drug (SD)	7.1 (1.3)	6.8 (1.4)	7.2 (1.8)	7.1 (1.6)
Median days of exposure to trial drug	7	6	7	7
Minimum	4	5	3	3
Maximum	10	9	12	12
Patients’ tolerability assessment, N (%)				
Very good	100 (92.6)	25 (83.3)	105 (64.8)	236 (78.7)
Good	7 (6.5)	5 (16.7)	50 (30.9)	64 (21.3)
Fair	1 (0.9)	0 (0.0)	6 (3.7)	7 (2.3)
Poor	0 (0.0)	0 (0.0)	0 (0.0)	0 (0.0)
Missing	0 (0.0)	0 (0.0)	0 (0.0)	0 (0.0)
Investigators’ tolerability assessment, N (%)				
Very good	101 (93.6)	26 (86.7)	109 (67.3)	236 (78.7)
Good	7 (6.5)	4 (13.3)	53 (32.7)	64 (21.3)
Fair	0 (0.0)	0 (0.0)	0 (0.0)	0 (0.0)
Poor	0 (0.0)	0 (0.0)	0 (0.0)	0 (0.0)

## Discussion

Observational studies play an important role in assessment of benefit/risk profiles of medicinal products, and they are often requested by health and regulatory authorities. Such studies add important information to results obtained in ‘classical’ phase III clinical trials [8]. A strength of the current observational study is that it was performed under real-life conditions in patients presenting from the community. Interpretation of the results is limited by the uncontrolled trial design; consequently, efficacy cannot be assessed in the strict sense. However, the study aimed to determine whether Rhinospray^®^ Plus benefited nonideal patients (that is, patients who administered the trial medication themselves in a home setting, including those with comorbid conditions and those who were pretreated or cotreated with other medications). The results indicate that this was so: for more than 95 % of patients—by both their own and the investigators’ assessments—effectiveness and tolerability were ‘very good’ or ‘good’. An improvement in at least one quality-of-life parameter was reported by 100 % of patients. The fact that no patient discontinued treatment is an additional indication of the acceptability of Rhinospray^®^ Plus.

Furthermore, the study included children and adolescents, who are commonly excluded from controlled clinical trials. When comparing data collected for the different age groups involved in the trial, effectiveness results (primary endpoints) were comparable between adults, children and adolescents. For quality of life parameters, even better results were observed in children and adolescents than for adults, especially with respect to quality of sleep. Similarly, treatment was even better tolerated by children and adolescents than adults.

Colds are self-limited illnesses; it may be argued that patients would experience symptomatic improvement with or without treatment over the time span of the trial. The current study was not designed to determine whether treatment with Rhinospray^®^ Plus shortened the duration of symptoms when compared with no treatment. However, literature comparisons suggest this may be so. In this trial, 51 % of adults, 54 % of children and 43 % of adolescents continued to report one or more nasal symptoms on day 7. This compares with a surveillance study in children in which 86 % of patients still had nasal symptoms after 6 days or more [9], and a prospective study in adults in which 72 % of patients still had at least one nasal symptom on day 7 [10]. It should also be noted that in the current study, more than 75 % of patients (all age groups) were free of nasal symptoms at the final visit (mean treatment duration: 7.1 days). These promising data should be further assessed in controlled clinical trials.

The results of this study indicate that Rhinospray^®^ Plus is well-tolerated and comfortable to apply. Community-based patients reported a relief in nasal cold symptoms and improvement in quality of life as a result of treatment.
